# Analytical Methods for Determination of Non-Nutritive Sweeteners in Foodstuffs

**DOI:** 10.3390/molecules26113135

**Published:** 2021-05-24

**Authors:** Viki Oktavirina, Nadhila B. Prabawati, Rohmah Nur Fathimah, Miguel Palma, Kiki Adi Kurnia, Noviyan Darmawan, Brian Yulianto, Widiastuti Setyaningsih

**Affiliations:** 1 Department of Food and Agricultural Product Technology, Faculty of Agricultural Technology, Gadjah Mada University, Jalan Flora No. 1, Bulaksumur, Sleman 55281, Indonesia; viki.o@mail.ugm.ac.id (V.O.); nadhilabenita@mail.ugm.ac.id (N.B.P.); r.nur.fathimah@mail.ugm.ac.id (R.N.F.); 2 Department of Analytical Chemistry, Faculty of Sciences, IVAGRO, Campus de Excelencia Internacional Agroalimentario (CeiA3), Campus del Rio San Pedro, University of Cadiz, Puerto Real, 11510 Cadiz, Spain; miguel.palma@uca.es; 3 Department of Marine, Faculty of Fisheries and Marine, Kampus C Jalan Mulyorejo, Universitas Airlangga, Surabaya 60115, Indonesia; kiki.adi@fpk.unair.ac.id; 4 Department of Chemistry, IPB University, IPB Dramaga, Bogor 16880, Indonesia; noviyandarmawan@ipb.ac.id; 5 Department of Engineering Physics, Institut Teknologi Bandung, Jl. Ganesha 10, Bandung 40132, Indonesia; brian@tf.itb.ac.id; 6 Research Center for Nanoscience and Nanotechnology (RCNN), Institut Teknologi Bandung, Jl. Ganesha 10, Bandung 40132, Indonesia

**Keywords:** high-intensity sweeteners, rapid analysis, extraction, food control, multianalyte analyses

## Abstract

Sweeteners have been used in food for centuries to increase both taste and appearance. However, the consumption of sweeteners, mainly sugars, has an adverse effect on human health when consumed in excessive doses for a certain period, including alteration in gut microbiota, obesity, and diabetes. Therefore, the application of non-nutritive sweeteners in foodstuffs has risen dramatically in the last decade to substitute sugars. These sweeteners are commonly recognized as high-intensity sweeteners because, in a lower amount, they could achieve the same sweetness of sugar. Regulatory authorities and supervisory agencies around the globe have established the maximum amount of these high-intensity sweeteners used in food products. While the regulation is getting tighter on the market to ensure food safety, reliable analytical methods are required to assist the surveillance in monitoring the use of high-intensity sweeteners. Hence, it is also necessary to comprehend the most appropriate method for rapid and effective analyses applied for quality control in food industries, surveillance and monitoring on the market, etc. Apart from various analytical methods discussed here, extraction techniques, as an essential step of sample preparation, are also highlighted. The proper procedure, efficiency, and the use of solvents are discussed in this review to assist in selecting a suitable extraction method for a food matrix. Single- and multianalyte analyses of sweeteners are also described, employing various regular techniques, such as HPLC, and advanced techniques. Furthermore, to support on-site surveillance of sweeteners’ usage in food products on the market, non-destructive analytical methods that provide practical, fast, and relatively low-cost analysis are widely implemented.

## 1. Use of Sweeteners in Foodstuffs and the Regulations

Sweeteners are originally derived from natural resources such as fruits and vegetables, while currently, a number of artificial sweeteners are available by chemical synthesis. These sweeteners are then distinguished into nutritive and non-nutritive sweeteners. The nutritive sweeteners supply energy (calorie), such as sugars, syrups, sugar alcohols or polyols, molasses, and honey. In comparison, the non-nutritive sweeteners provide no or very low amounts of energy (low-calorie sweeteners), such as aspartame, acesulfame-k, neotame, saccharin, sucralose, and cyclamate [1,2].

Most of the processed foods are prepared with both nutritive and non-nutritive sweeteners added to the ingredients. Although the nutritive sweeteners are considered as Generally Recognized as Safe (GRAS), consumption of these sweeteners, mainly sugars, has an adverse effect on human health when consumed in excessive doses for more than a decade, including alteration in gut microbiota, obesity, and diabetes [3,4]. Imamura et al. [5] have conducted a meta-analysis and survey analysis of the effects of consumption of sugar-sweetened beverages on type 2 diabetes. The results show that consumption over ten years is associated with the incidence of type 2 diabetes. 

Therefore, the application of non-nutritive sweeteners in foodstuffs has risen dramatically in the last decade to substitute sugars. These typical sweeteners are commonly recognized as high-intensity sweeteners because, in a lower amount, they could achieve the same sweetness of sugar. Because of this reason, non-nutritive sweeteners have been widely used in industries to prepare various foods claimed as “diet” or “light” products. These relatively low-calorie products are attractive to consumers who are maintaining body weight or controlling blood sugar in the management of diabetes. Besides, non-nutritive sweeteners are safe for oral health because they are not fermented by microorganisms that cause dental plaque [3].

Regulatory authorities and supervisory agencies worldwide have established the maximum amount of non-nutritive sweeteners used in food products. The right consumption of non-nutritive sweeteners is beneficial over sugars for energy intake; however, prolonged and high consumption of these sweeteners could lead to some adverse effects on human health. Long term intake of aspartame can change antioxidant defense status and histopathology in the liver [6], while acesulfame-k can also cause damage to DNA [7]. Chi et al. [8] reported neotame effect on the gut microbiome, and it concluded that neotame induces adverse effects on gut microbiota. The risk of obesity and diabetes also increased due to long-term saccharin consumption [9]. 

Some regulatory authorities deal with food control around the globe, such as Joint FAO/WHO Expert Committee on Food Additives (JECFA), Codex Alimentarius Commission (CAC), Food and Drug Administration (FDA) from the United States, Food Standards Australia New Zealand (FSANZ), and National Agency of Drug and Food Control (NADFC) of the Republic of Indonesia have determined the Acceptable Daily Intake (ADI) value and maximum amount for high-intensity sweeteners in foodstuffs (Table 1). In contrast, there is no labeling regulation for these sweeteners.

While the regulation is getting tighter on the market to ensure food safety, reliable analytical methods are required to assist the surveillance in monitoring the use of high-intensity sweeteners. Reliable detection and quantification of non-nutritive sweeteners are mandatory for an immense range of food matrices to ensure food safety. Occasionally, rapid detection is necessary for on-site inspection. 

**Figure 1:**
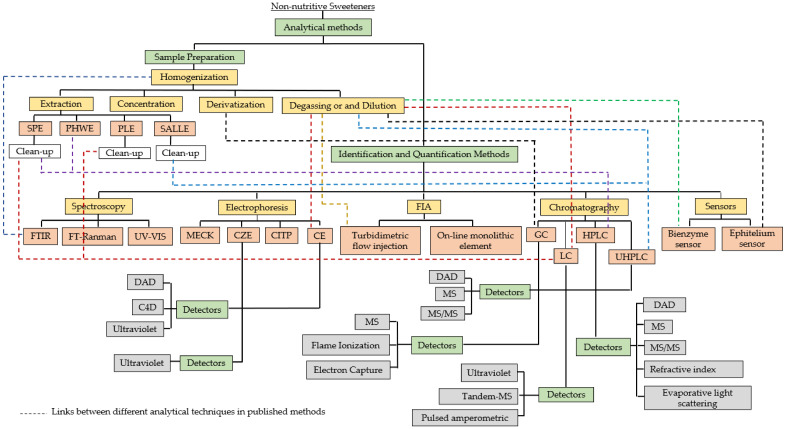
Diagram of the analysis method of non-nutritive sweeteners in food matrices.

There are numerous of analytical methods available for the determination of non-nutritive sweeteners. Depending on the type of food matrices, the instrument used, and the desired degree of accuracy, various approaches for sample preparation are proposed. Figure 1 describes an outline of the methods employed for non-nutritive sweeteners analyses on food matrices. 

## 2. Sample Preparation in the Analysis of Non-Nutritive Sweeteners 

The primary step in most analytical methods is sample preparation, that allows for the sample to be suitable for the later analytical steps. It is essential when dealing with complex matrices of food samples containing fats, proteins, dyes, preservatives, vitamins, and minerals [3]. In this case, a method of separation or purification of non-target compounds is required to remove other compounds affecting the analytical signal. Prior to the analysis of non-nutritive sweeteners, extraction is frequently performed for sample preparation, mainly for solid food samples. Moreover, for samples containing a low levels of the sweeteners, additional sample preparation step such as clean up and concentration is required after the extraction process [10]. The proper procedure, efficiency, and the use of solvents are considered in selecting a suitable extraction method for a specific food matrix. 

For solid samples, especially plant materials such as stevia leaves, a prior extraction step is needed to guarantee full recovery of the sweeteners from the that kind samples, because of dissolving them without advanced treatment does not guarantee a reliable determination. More efforts are required to damage cells or tissues to facilitate diffusion and interaction between analytes and solvents. This sample treatment is also applied to other similar samples. Meanwhile, in food products, the extraction process may be simpler than in raw materials. Solid food products such as candy, cake, jelly, and various canned fruit can be extracted by using less power consumption. In addition, liquid samples, such as various juices can be treated by using centrifugation to separate undissolved solids, while the air bubble of soft drinks can be removed by using sonication.

Moreover, for samples containing low levels of the sweeteners, the sample preparation step must also include some concentration procedures to guarantee a reliable determination. These sample preparation steps apply to both solid and liquid samples.

Conventional extraction methods such as Soxhlet, reflux, sonication, and liquid–liquid extraction (LLE) have been widely applied in food analysis. However, fast and practical sample preparation is currently preferred, especially for the application in food industries or by the regulatory authorities. Thus, the development of extraction methods such as supercritical fluid extraction (SFE) [11], microwave-assisted extraction (MAE), pressurized liquid extraction (PLE) [12], subcritical water extraction (SBWE), and pressurized hot-water extraction (PHWE) [13] has been proposed by researchers. These modern extraction techniques are also known as the green solvent extraction method, with fast and high reproducibility [3].

PHWE method, for instance, has been used to extract steviol glycosides (stevioside and rebaudioside A) from *Stevia rebaudiana* Bertoni (sugar leaf). The significant advantage is the use of water as the extraction solvent, fast, and reduced energy of the process that leads to a lower cost. The use of pressurized water in PHWE under conditions of high temperature and controlled pressure can increase the mass transfer yet maintain the stability of bioactive components such as steviol glycoside [13]. Yildiz-Ozturk et al. [14] conducted a study, using SBWE to extract steviol glycosides by using a sample-to-water ratio of 1:10 (*w*/*v*). When water is used as a solvent in PLE, the extraction process is also known as PHWE with conditions below the supercritical point, that is, <374 °C and pressure of 218 atm [15].

Apart from PHWE, PLE is also applied routinely at analytical laboratories as an extraction technique. The utmost distinction between PHWE and PLE lies in the use of solvent. PLE uses a various types of solvents, both single or a mixture of solvents such as alcohols or alkanes, whereas PHWE merely employs water as the extraction solvent. Additionally, the working temperature of PLE is above the boiling point of the solvent used up to 200 °C [15], whilst PHWE could reach 374 °C (the critical temperature for water) [16]. Because PHWE uses water solely as the solvent, only the most polar compounds are extracted from the samples; then usually, it does not require a clean-up method to recover analytes [17]. Meanwhile, for an application in non-nutritive sweeteners analysis, PLE with solvents less polar than water usually also extract some less polar compounds than sweeteners, then it requires a clean-up process such as solid-phase extraction [18]. A further cleaning step has been reported to be effective in removing impurities from food matrices [16].

SPE is one of the most popular sample preparation techniques used to extract or clean-up samples necessary for an analysis method. It is effectively applied to analyze target compounds in liquid form matrices [10,19]. Because foods are complex matrices, SPE is frequently applied in the purification step for extracts or liquid samples. A typical SPE, like a dispersive SPE, can be used as an alternative to Gel-Permeation Chromatography [12]. 

SPE consists of a solid sorbent part contained in a device called a cartridge. The most common types of SPE cartridges, such as C_18_ silica-based, phenyl-bonded silica, and reversed-phase polymeric sorbents [20]. Adjusted to the characteristics of the target analyte, the copolymer sorbents based on divinylbenzene/hydrophilic N-vinylpyrrolidone, PS-DVB (hydroxylated polystyrene/divinylbenzene, SDVB (styrene/divinylbenzene, SDVB), and PWAX (polymer weak anion exchange) have been used for the analysis of non-nutritive sweeteners in complex aqueous samples [19].

Sep-Pak C_18_ cartridge was successfully used to purify the analytes of non-nutritive sweeteners from complex matrices such as chocolate and dairy products that have a high fat content [21]. The cartridge is also applied for the analysis of non-nutritive sweeteners such as aspartame, acesulfame-K, saccharin, and cyclamate in fermented milk drinks and preserved fruit [22]; cyclamate in beverages, syrup, and jam [23]; erythritol, xylitol, dulcin, alitame, maltitol, neotame, sucralose, neohesperidin dihydrochalcone, stevioside, and rebaudioside A in extracts from hard candies, carbonated and non-carbonated drinks, and yogurt [24].

The SPE mechanism of action includes several stages. First, the cartridge is activated with a specific solvent, then the sample solvent is used to create a pH equal to the sample to avoid unexpected chemical changes. Subsequently, then the sample is added and usually the analyte from the sample solution is retained by the solid phase, then washed (also called the rinsing step) to remove interference in the matrix from the analyte by a selective washing solution. The final step is elution, the removal of desired analyte from the sorbent by using a solvent with high affinity to the analyte. The resulting SPE extract can then be introduced into the determination system [25]. 

There are different types of interaction in the SPE technique such as polar stationary phase, non-polar stationary phase, and ion-exchange SPE (anion–cation interaction). When applying normal phase SPE to extract polar analytes, an elution stage is required, as previously described. However, when applying the reverse phase to a polar analyte, the elution process is not required because the polar analyte will pass directly. At the same time, the non-polar contaminants will be retained by the absorber [26]. This advantage is suitable for the analysis of non-nutritive sweeteners because they are polar.

To increase the sensitivity and selectivity in sample analysis, the SPE method assists other analytical methods such as Gas Chromatography (GC–MS), HPLC, and capillary electrophoresis (CE) (Table 2). Importantly, to increase accuracy and precision, SPE can also be automated [15].

In order to endorse the development of a rapid analysis method, some samples can be analyzed directly or with minimal pretreatment [3]. More practical or fewer processing steps in sample pretreatment could reduce the analysis time, also increasing the reproducibility. A minimal pretreatment process includes at least dissolution, degassed by sonication, and filtering [27,28]. This approach can be applied to several types of food samples such as sauce, jam, instant beverages, nectar, and ready to drink products [29,30,31]. Apart from converting a food matrix into a sample suitable for analysis, pretreatment is also aimed to improve the analytical method, especially for those that require high sensitivity.

In addition to the aspect of time efficiency, the selection of sample preparation techniques must also be adjusted to the analytical method used. For example, the use of Gas Chromatography (GC) for non-nutritive sweeteners analysis requires a derivatization process. This is because sweeteners have low volatility, and so they must priorly be converted into derivative products that are more volatile. Or because a specific detection system is going to be used, for example, Electron Capture Detector [3].

The derivatization process has been applied to analyze cyclamate by GC in food and beverage samples. The non-volatile cyclamate was converted into a volatile compound of *N,N*-dichlorocyclohexylamine, using sodium hypochlorite. Subsequently, the derivatization product can be eluted in the GC system, later it can be detected by Electron Capture Detector [32], or other detection systems. Apart from sodium hypochlorite, the derivatization process by chlorine compounds is also an alternative approach to form *N,N*-dichlorocyclohexylamine [33]. Cyclamate could also be analyzed by GC in the form of cyclohexylamine through hydrolysis by acid or alkaline, including nitric acid. This approach allows for the compounds to be determined by GC because of the higher volatility of the new chemical forms after derivatization [34].

Each analyte of non-nutritive sweeteners has a specific derivatization process prior to a GC analysis. In most cases, to increase the volatility, an esterification procedure is required, for example, for saccharin analysis, and sucralose is firstly silylated before the analysis, whereas aspartame and dulcin analysis can be carried out without derivatization process. The latter compounds are volatile enough to be analyzed by using GC [3].

The sample preparation step is undesirable for some purposes, mainly when the analysis should be performed rapidly or in real-time. Non-destructive analytical methods that do not require sample preparation steps, such as simple dissolution, provide an advantage in terms of analysis time efficiency. These rapid methods are fit for on-site inspection to control the use of non-nutritive sweetener or suitable to assist the quality control in food industries. A further review on rapid determination for sweeteners in foods is provided in the last section.

**Table 2:** Sample preparation for non-nutritive sweeteners in some food matrices.

Analyte	Matrix	Extraction Method	Sample: Solvent Ratio	Solvent	Extraction Conditions	Determination Method	Reference
Bioactive compounds and steviol glycosides	*Stevia rebaudiana* leaves	PHWE	2:7.5	Distilled water	Temperature 160 °C; static 5 min; extraction cycle 2; pressure 103.4 bar; flushing 60%	HPLC–UV	[13]
Stevioside, rebaudioside A	*Stevia rebaudiana* leaves	Supercritical CO_2_ extraction	1:44	CO_2_ 99%	Pressure 200 bar; temperature 30 °C; extraction time 12 h	HPLC–UV/Vis	[11]
Rebaudioside A	*Stevia rebaudiana* leaves	Supercritical CO_2_ extraction	1:30	CO_2_ 99%	Temperature 80 °C; pressure 211 bar	HPLC–UV	[35]
				CO_2_ + co-solvent 17.4% ethanol in water			
Acesulfame-K, saccharin-Na, aspartame, benzoate-Na, sorbate-K	Juices	SALLE	6:1	ethanol:acetone (50:50)	pH adjustment to 3 with HCl solution (0.7 M, *v*/*v*); ammonium sulfate to complete the dissolution of salt	UPLC–UV	[36]
Cyclamate	Fruit in syrup, jam, orange juice, *shokosyu*, pickles, confectionery, soy sauce, sunflower seeds, and *waume* (diluted in 50 mL of 0.1 mol/L hydrochloric acid)	SPE	6:1	Demineralized water and 50% aqueous methanol (1:1)	Oasis HLB cartridge; conditioning: methanol and demineralized water (10 mL each); rinsing: demineralized water, 50% aqueous methanol (2 mL each).	CE	[23]
Acesulfame, cyclamate, saccharin, aspartame, sucralose, neohesperidin dihydrochalcone, neotame	Wastewater, tap water, surface water (including river water and seawater), and groundwater	SPE	8:1	Methanol containing 1 mM tris (hydroxymethyl) aminomethane	Poly-Sery PWAX cartridge; conditioning: methanol, 25 mM acetic acid–sodium acetate, and buffer at pH 4 (6 mL each); rinsing: buffer at pH 4 (6 mL); flow rate of 1 mL/min	HPLC–MS/MS	[37]
Acesulfame-K, aspartame, sucralose, rebaudioside A	Hard candies and carbonated beverages (dissolved and diluted 50-fold in water) Yogurt (dissolved in 50 mL of 0.075% formic acid + 3 mL DIPEA and diluted 25-fold in water)	SPE	1.25:1	Methanol	C18 cartridge; buffer: 0.075% formic acid + DIPEA adjusted at pH 4.5 conditioning: 1.5 mL of methanol, 3 mL buffer; rinsing: 1.5 mL buffer	UHPLC–MS/MS	[24]
Acesulfame-K, alitame, aspartame, cyclamate-Na, glycyrrhizic acid, neotame, neo-hesperidin dihydrochalcone, saccharin-Na, stevioside, sucralose	Fish	PLE–SPE	1:17 5:1	PLE, methanol:water (1:1) SPE, methanol	Pressure 103.4 bar; preheating 5 min; cycle 1; temperature 60 °C; static time 5 min; flushing volume 50%; purge 300 s. Oasis HLB; Conditioning: methanol, water at pH 3 with formic acid (5 mL each); rinsing: 5 mL water:methanol (9:1; *v*:*v*)	LC–HRMS	[12]

PHWE, pressurized hot-water extraction; SPE, solid-phase extraction; PLE–SPE, pressurized liquid extraction–solid-phase extraction; SALLE, salting out liquid–liquid extraction; HPLC–UV/Vis, High-Performance Liquid Chromatography–Ultraviolet/Visible; UHPLC–MS/MS, Ultrahigh-Performance Liquid Chromatography–Tandem Mass Spectrometry; UPLC–UV, Ultra-Performance Liquid Chromatography–Ultraviolet; LC–HRMS, Liquid Chromatography–High-Resolution Mass Spectrometry; GC–ECD, Gas Chromatography–Electron Capture Detector.

Apart from foods produced by industries, non-nutritive sweeteners are also found in several types of water [38]. There is a safe limit for non-nutritive sweeteners in water that does not have adverse effects on aquatic organisms, for example, sucralose, which shall not be more than 1000 mg L^−1^ [12,38,39]. However, it turns out that non-nutritive sweeteners are also reported to be identified in several fish species, such as striped red mullet (*Mullus surmuletus*) and common carp (*Cyprinus carpio*). The levels of the sweeteners in both fishes were ranged from 12.5 to 250 ng g^−1^ (d.b.). Because of that, in the framework of analytical development, non-nutritive sweeteners have been classified as emerging organic contaminants (EOCs) in the last few decades [12]. EOCs were studied because they may have adverse effects on health and the ecosystem [40]. However, this idea still needs to be highlighted because the information is yet limited, including toxicological studies of aquatic organisms.

## 3. Conventional Methods for Non-Nutritive Sweeteners Determination

Over a couple of decades, there has been an increased interest in using various analytical techniques for the identification and quantification of non-nutritive sweeteners such as aspartame, saccharin, cyclamate, acesulfame-K, and sucralose in various food matrices. Nowadays, a method capable to simultaneously determine multi-sweeteners in a single analytical run is required to assist the food-safety monitoring, provided that a combination of non-nutritive sweeteners is ubiquitous in food products.

Some chromatographic techniques are widely dedicated to determining the non-nutritive sweeteners, including Gas Chromatography (GC), High-Performance Liquid Chromatography (HPLC), Ion Chromatography (IC), Micellar Electrokinetic Chromatography (MEKC), and Thin-Layer Chromatography (TLC). In comparison, spectroscopic techniques are proposed for a faster analytical method such as UV/Vis Spectroscopy, Fourier-Transform Infrared Spectroscopy (FTIR), and Fourier-Transform Raman spectrometry. Other advanced techniques are also available, i.e., capillary electrophoresis (CE) [10], capillary zone electrophoresis (CZE) [41,42], and flow-injection analysis (FIA) [43,44,45,46]. 

Among the aforementioned analytical methods, HPLC is the most prevalent technique for multianalyte analysis in industries and analytical laboratories. The fundamental principle of HPLC separation is based on the different affinities of analytes to the stationary phase column and to the mobile phase. Compounds with higher affinity to the stationary phase will be stronger retained in the column and separate from those with lower affinities. Hence, HPLC can be used to analyze multiple analytes merely in a single run by optimizing the gradient of the mobile phase [47].

The identification and quantification of non-nutritive sweeteners by HPLC must be assisted with a suitable detector. Some alternative detectors include UV/Vis detector, usually as diode-array detector (DAD), refractive index (RI) detector, Mass Spectrometry (MS), light scattering, and conductivity detector [3,28,48]. However, in some cases, the detection method should be improved to reach a reliable determination. For instance, in cyclamate and sucralose analysis, the interaction between the analyte with derivatization reagents is needed before injection into HPLC with UV detectors. Sucralose has a low absorptivity molar coefficient at most wavelengths in the UV range. To increase the sensitivity of sucralose to UV/Vis absorption, it must be derivatized by using *p*-nitrobenzoyl chlorine (PNBCI) reagent. Whilst cyclamate can be reacted with diazomethane, chlorine, or *N*-heptafluorobutiryl anhydride [34]. By this approach, both analytes can be then detected and quantified by the UV/Vis detector. 

The fastest version of analysis using Liquid Chromatography techniques is Ultra-Performance Liquid Chromatography (UHPLC). The separation occurs inside a column packed with smaller particles (<2 µm) than the regular HPLC. Hence, the UHPLC system should be supported by higher pressure to achieve fast separation with superior resolution and sensitivity [49]. Since the analysis time is faster than the regular HPLC method, UHPLC consumes a significantly lower amount of solvents and generates a lower waste. UHPLC coupled with diode array detector provided excellent resolution for 11 min simultaneous separation of acesulfame-k, saccharin, cyclamate, and aspartame in nine food matrices (soft drink, nectar, juice, ready to drink tea, jam, barbeque sauce, tomato sauce, instant pudding, instant juice) [29]. Unfortunately, cyclamate has very low absorbance to UV–Vis or diode array detectors. Therefore, a derivatization step is required to enhance the signal.

Cyclamate can be converted into *N,N*-dichloro-cyclohexylamine by sodium hypochlorite; then, it can be determined at 314 nm [33]. In this form, cyclamate also has a substantial electronegativity property, resulting in rapid detection by GC coupled with Electron Capture Detector (GC–ECD). It took less than 6 min to detect cyclamate in yellow wine, cake, fruit juice drink, and preserved fruit by GC–ECD [32].

To achieve a more practical analysis, an alternative method without derivatization is available for cyclamate employing a different detection system. Cyclamate in beverage and jam can be measured without derivatization by using the electrophoresis method, specifically CE [23,50]. The UV-absorbing electrolyte is added with a cationic surfactant to detect cyclamate selectively. Hence, a capillary electrophoresis method coupled with indirect UV detection can be applied as an alternative for cyclamate analysis [51]. Additionally, Pacakova and Stulık, [52] reported the advantages of using CE over HPLC for fewer sample volume, automated, better resolution, more practical, faster, and environmentally friendly. Subsequently, some researchers succeeded in identifying non-nutritional sweeteners, such as aspartame, cyclamate, acesulfame k, and saccharin, in the food matrix by using the CE method [53,54,55,56].

Capillary zone electrophoresis (CZE) is the simplest and most commonly applied CE mode in electrophoretic separation techniques that separates the analytes based on the difference in the velocity of charged particles. This velocity is measured based on electroosmotic flow and electrophoresis mobility. Apart from the movement of negative and positive ions to the opposite electrode, the ion with a small size is faster towards the electrode than the larger ion size. Inversely, the ion with a large charge (cation) will move to the detector faster than the anion charge. At the same time, neutral particles will be in a stationary condition to form a sample zone [57,58]. Once a high-power voltage is applied into the neutral zone, all particles move towards the cathode and detector; thus, the compounds can be separated completely. 

Recently, simultaneous separation and detection of aspartame, cyclamate, acesulfame-K, and saccharin in soft drinks, liquid and solid sweeteners, peach tea, lemon tea, and syrup has been reported, using CZE–indirect UV detection at 220 nm [42]. Cyclamate quantification by CE with indirect UV is an hour faster than the HPLC separation [23]. Even faster, the detection of sucralose through the use of CE can be performed merely in 16 min [59]. Furthermore, when the CE is combined with Capacitively Coupled Contactless Conductivity Detection (C4D), analysis of aspartame, cyclamate, saccharin, and acesulfame-k can be performed less than 6 min [60].

Another method that can be used as an alternative in the analysis of additives, especially non-nutritive sweeteners, is flow-injection analysis (FIA). As sample preparation is omitted, this method can be developed as a rapid measurement for a large number of beverages samples [3]. For example, 40 measurements can be performed within an hour because the single FIA analysis of aspartame in soft drinks was merely performed in 1.5 min including baseline stabilization, sample injection, recording, and washing [61]. This method minimizes the use of hazardous solvents to analyze the micro quantities sample, thus generating less waste. Hence, FIA is considered environmentally friendly and has been widely reported as a green analytical method [62]. In addition, FIA is widely incorporated with spectrophotometric detection, such as UV or DAD [3]. 

In the case of cyclamate analysis by UV detection, a derivatization step is required. However, most of the derivatization techniques are time-consuming and require hazardous chemicals, thus making them incompatible with the principles of environmental friendliness. Several green alternatives are offered as a substitute for derivatization techniques such as headspace solid-phase microextraction (HS-SPME) [63], solid-phase microextraction (SPME), single-drop microextraction (SDME), head-space single-drop microextraction (HS-SDME), etc. [64]. Unfortunately, cyclamate analysis using the microextraction method still utilizes sodium nitrite and sulfuric acid [34] that are harsh chemicals. In large quantities, these chemicals can pollute the environment. Hence, the claim as a green alternative is most likely seen from using a significantly reduced amount of derivatization reagent than conventional techniques.

Due to the limited information that supports cyclamate analysis using an FIA coupled with a UV detector, further discussion is needed, especially the suitable sample preparation method considering the potential of FIA as a reliable technique for routine analysis.

Besides providing a more practical procedure, sample preparation prior to analysis is preferably omitted to avoid analytical error due to additional analysis steps. Vibrational spectroscopy as a non-destructive technique, including FTIR and FT-Raman, proposes a direct qualitative and quantitative determination of non-nutritive sweeteners in solid samples. The analytical methods based on these techniques usually do not need sample preparation steps [65,66].

Employing FTIR, the presence of non-nutritive sweeteners can be qualitatively detected by determining the functional group so that the structure of target analytes can be specified. To further quantify the analyte, the concentration of the non-nutritive sweeteners is calculated from interpolation in a standard curve made from a series of known concentrations of standard compounds. Five different sweeteners (sodium cyclamate, acesulfame-K, aspartame, sucralose, and sodium saccharin) were successfully identified and quantified by using FTIR in a diet tea [66]. Meanwhile, sodium cyclamate and saccharin in commercial tabletop sweeteners available on the market were successfully measured by using FT-Raman [67].

Raman and FTIR spectroscopy differ in several fundamental ways. Raman spectroscopy measured the frequency of light scattered by the sample, while FTIR records the amount of light absorbed [68]. In brief, the main difference between these two methods is the susceptibility of the wavelengths at which they operate. FTIR has a wider recording area than Raman, i.e., 450–4000 cm^−1^ [69], while the fingerprint scanning of Raman is ranged from 800 to 1800 cm^−1^ [70] and requires software for assisting the data processing [71]. Both spectroscopy methods detect vibrations. IR depends on the change in dipole moment, while Raman is on molecular polarizability [68]. However, when combined, these two methods are powerful tools for material characterization.

Duarte et al. [72] reported that the Raman technique was successfully employed for multi-analyte analysis. Four types of non-nutritional sweeteners, such as aspartame, cyclamate, saccharin, and acesulfame-K in table sweeteners, can be determined by Raman spectroscopy with simple sample preparation. This approach is also best suited to food industries as this analytical tool can be automated [65,73]. 

For more detailed information such as the condition of the instrument, the advantages and disadvantages of the analysis conducted by previous researchers can be seen in Table 3.

## 4. Rapid Methods for Non-Nutritive Sweeteners Determination

The usage of non-nutritive sweeteners must respect the general legislation in force in the country and requires a rapid yet reliable analytical method for surveillance. Hence, new analytical methods that require minimal or no sample preparation step, practical, low-cost, and fast are proposed. Some rapid determination methods were developed on the basis of advanced analytical techniques, such as electrophoresis (CE), chromatography (UHPLC), vibrational spectroscopy (FT-NIR), and sensor and biosensor combined with chemometrics (Table 4).

Chemometrics is a statistical approach method that has been widely used to solve analytical problems. For instance, in chromatography, some analytical problems such as retention time shifts, overlap, undesirable background signals, and data compression can be overcome with the aid of chemometrics. Furthermore, in spectroscopy, spectrum noise and eliminating the non-significant variables can be achieved by chemometrics [88]. These statistical tools are also helpful in optimizing the combination of the selected intervals and conducting comparisons among the prediction performance of local models and full-spectrum models. Least Squares (LS), Partial Least Squares (PLS), and Principal Component Regression (PCR) are approaches that can be applied to multicollinearity problems. In brief, compared to PCR, the PLS technique gives better results in solving a large number of independent variables [89]. These statistical approaches help in gather information and speed up the processing of analytical data. Hence, chemometrics is a great tool to support the development of rapid analytical methods when some analytical problems or big data are acquired [90].

Besides providing rapid analyses, particular analytical methods offer simplicity based on vibrational spectroscopic techniques such as FTIR, FT-NIR, and FT-Raman. These methods permit the analysis of large sample quantities in solid form. Even samples can be analyzed without being removed from the packaging as long as the packaging is visually transparent. Despite these advantages, overlapping or low-quality spectra often occur in the analysis of complex samples. However, the use of multivariate data analysis, such as Partial Least Square (PLS), Interval PLS (iPLS), Synergy Interval (siPLS), Principal Component Regression (PCR), or Counter-Propagation Artificial Neural Networks (CP-ANN), has been proven to improve the analysis results [65,72,91].

Another technique for rapid analysis is the voltammetry method that is classified as an electroanalytic or electrochemical method for substance analysis. The voltammetry method can be used to determine the concentration of analytes directly without or with minimal pretreatment, analyze colored materials in samples and dispersed solid particles, and determine the concentration of several analytes simultaneously [92]. For example, linear-sweep voltammetry with a rotating disk electrode (RDE) assisted with the PLS algorithm has been successfully tested to analyze acesulfame-k and aspartame in sweeteners powder [93]. Square-wave voltammetry with a Boron-Doped Diamond (BDD) electrode was found to be effective for simultaneous determination of aspartame and cyclamate in dietary products [92]. Another voltammetric method using Screen-Printed Carbon Electrode (SPCE) coated by caffeic acid polymer film can also determine aspartame in carbonated commercial drinks [94]. These voltammetric methods only require degassing and diluting the liquid samples. If the samples were in solid form, they were dissolved first [92,93,94].

The latest version of the current rapid method utilizes electronic tongue, taste sensors, and even biosensors. Electronic tongue (e-tongue) is a multisensor system that mostly uses metal and ion-selective electrodes in voltammetric measurements. Principal Component Analysis (PCA) was used to assess the different potential responses and then discriminate foods based on the studied analytes. Moreover, the taste sensor used a lipid or polymer membrane on its electrodes. The developed taste (sweetness) sensor had satisfactory performance during the tests [95]. It can obtain a response as low as 10 mM aspartame, no response to other basic tastes, and concentration dependence on aspartame.

**Table 4:** Rapid methods for the determination of sweeteners in food products.

Analyte	Matrices	Analytical Method	Chemometrics	Sample Preparation	Analysis Time	Reference
Acesulfame-k, Saccharin, Cyclamate, Aspartame, Neotame	Ready to drink tea, soft drink, nectar, instant juice, instant pudding, jam, barbeque sauce, tomato sauce	UHPLC–Diode Array Detector	Multivariate using central composite design	Dilution, degassing, and centrifugation if necessary	10 min	[29]
Aspartame, Cyclamate, Saccharin, Acesulfame-k	Soft drinks and tabletop sweetener formulations	CE with C4D	Linear regression	Degassing and dilution	6 min	[60]
Aspartame, Acesulfame-k, Cyclamate, Saccharine, Phenylalanine	Drinking water	SPE–LVSS–CE	Linear regression	Clean-up by SPE	4 min	[10]
Saccharin, Acesulfame-k	Sweeteners (powder, liquid, tablets), fruit juices powder	UV–Vis Spectrophotometry coupled	PLS-1	Dissolving in an appropriate pH and solvent	<10 min	[96]
Aspartame, Acesulfame-k	Powder commercial sweeteners	UV Spectrophotometry	PLS-2	Dilution	<10 min	[91]
Saccharin, Cyclamate	Tabletop sweeteners	Vibrational spectroscopy based on Raman and NIR	PLS	Direct measurement	<10 min	[91]
Aspartame	Powder tabletop sweeteners	FT-Raman Spectroscopy	PLS, PCR, CP-ANN	Sample homogenization	<10 min	[65]
Acesulfame-k, aspartame	Powder sweeteners	Linear sweep voltammetry	PLS	Dilution	40 s	[93]
Aspartame	Soft drinks	Cyclic voltammetry using Screen-Printed Carbon Electrode	Linear regression	Degassing and dilution	<10 min	[94]
Aspartame, cyclamate	Powder juice, carbonated guarana drink	Square-wave voltammetry using a Boron-Doped Diamond electrode	Linear regression	Dilution	<10 min	[92]
Saccharin	Dietary sweeteners	Flow-injection analysis system (turbidimetric) using UV–Vis Spectrophotometer	Multivariate using Doehlert design	Dilution in deionized water	8 min	[97]
Aspartame, Acesulfame-k, Saccharin	Foods and soft drinks	Flow-injection analysis using spectrophotometer DAD	Linear regression	Dilution and centrifugation if necessary	10 min	[98]
Saccharin, Acesulfame-k	Liquid sweeteners	Sweetness sensor membranes	N/A	N/A	N/A	[95]
Saccharin, Cyclamate	Carbonated drink	Biosensor using intact taste epithelium	-	Degassing	7 s	[87]

C4D, Capacitively Coupled Contactless Conductivity Detection; SPE–LVSS, solid-phase extraction–large volume sample stacking; UV–Vis, Ultraviolet–Visible; PLS, Partial Least Square; iPLS, Interval PLS; siPLS, Synergy Interval PLS; PCR, Principal Component Regression; CP-ANN, Counter-Propagation Artificial Neural Networks.

Interestingly, not only lipid polymer membranes can be used as sensors in electrodes, but also cells and tissues, which make them called biosensors. Taste bud from epithelium was isolated and placed on the surface of the microelectrode array (MEA), so the saccharin and cyclamate in sweetened carbonated drinks can be detected [87]. When using sensors, if the samples are in liquid form, the sample preparation is minimal and considered a non-destructive technique. Nevertheless, if the samples are in solid form, it is needed to priorly perform an extraction. Generally, the results of the analysis by sensors can be found in seconds [87,95].

## 5. Methods

### 5.1. Data Sources

This review was prepared on the basis of literature published from February 2000 to January 2021 indexed by Scopus, Google Scholar, and ScienceDirect. The information was collected from reviews and research articles. Keywords for searching the relevant information were as follows: non-nutritive sweeteners; non-nutritive sweeteners in food; analytical methods for determining non-nutritive sweeteners in food; and rapid methods for non-nutritive sweeteners analysis. The data collected were divided into several points, i.e., sample preparation, identification and quantification, and rapid methods. 

### 5.2. Inclusion and Exclusion Criteria

The data collected included the following: (a) sample matrix and type of sweeteners; (b) sample preparation, including extraction method, solvent used, sample to solvent ratio, and extraction conditions; (c) instruments and conditions used, including evaluation of advantages and disadvantages; and (d) chemometric techniques. Exclusion criteria were as follows: published in non-English language; analytes were nutritive sweeteners or sugars.

## 6. Conclusions

This systematic review presents the alternative analytical methods for non-nutritive sweeteners in foodstuffs from numerous studies reported from February 2000 to January 2021. There are different analytical methods to determine non-nutritive sweeteners in various food matrices. The selection of the analytical methods, including extraction, separation, and detection, depends on the characteristic of sample matrices and target analytes. Each method has its advantages and disadvantages. A significant advantage was the ability to perform simultaneous analysis for multianalyte of non-nutritive sweeteners while achieving an excellent method validation result. Moreover, the existence of a fast extraction and separation method provides high effectiveness for the analysis. However, some drawbacks also appear, providing non-satisfactory analysis results. For example, direct UV detection is not recommended for analytes that do not have a chromophore group. To overcome the disadvantage related to the low sensitivity of the method, extra steps of sample pretreatment are needed, depending on the sample characteristics. The appropriate analytical methods supported the attempt to assist the surveillance in monitoring the use of high-intensity sweeteners available on the market. Reliable detection and quantification of non-nutritive sweeteners are mandatory for an immense range of food matrices to ensure food safety.

## Figures and Tables

**Table 1:** Acceptable daily intake and the maximum usage of high-intensity sweeteners in food defined by selected regulatory authorities.

Sweeteners	Acceptable Daily Intake		Maximum Usage in Food	
	Dose (mg/kg Body Weight/Day)	Regulatory Authorities	Dose (mg/kg Product)	Regulatory Authorities
Aspartame	0–40	JECFA, FSANZ	500–5500	CAC
	0–50	FDA, NADFC	150–10,000	FSANZ
Acesulfame-k	0–15	JECFA, FSANZ, FDA, NADFC	200–1000	CAC
			200–3000	FSANZ
Advantame	0–32.8	FDA	3–100	CAC
	0–5	JECFA, FSANZ		
Neotame	0–2	JECFA, FSANZ, NADFC	2–1600	FDA, FSANZ
Saccharin	0–5	JECFA, FSANZ, NADFC	80–5000	CAC
Sucralose	0–15	JECFA, FSANZ, NADFC	120–5000	CAC
Calcium Cyclamate	0–11	JECFA, FSANZ, NADFC	100–2000	CAC
Cyclamic Acid	0–11	JECFA, FSANZ, NADFC	100–2000	CAC

Note: Joint FAO/WHO Expert Committee on Food Additives (JECFA), Codex Alimentarius Commission (CAC), Food and Drug Administration (FDA), Food Standards Australia New Zealand (FSANZ), and National Agency of Drug and Food Control (NADFC).

**Table 3:** Analytical methods for simultaneous determination of non-nutritive sweeteners in different foodstuffs.

Sweeteners	Sample Matrix	Method	Analysis Condition	Advantages	Drawbacks	Method Characterization	Reference
Acesulfame, saccharin, and aspartame	Juices	RP-UHPLC–UV	Acquity UPLC BEH C18 (100 mm × 2.1 mm, 1.7 µm) column; UV detection at 210 nm; mobile phase A: ammonium acetate with 0.01% of trifluoroacetic; mobile phase B: acetonitrile; gradient elution; flow rate: 0.2 mL/min; column temperature: 40 °C; injection: 10 µL	Acceptable recovery; fast separation (less than 6 min)	Requires ultrahigh-pressure equipment of chromatography	Recovery: 84.97–122% LOD: 0.3–1.42 mg/L LOQ: 0.99–5.14 mg/L	[36]
Acesulfame, saccharin, cyclamates, aspartame, sucralose, alitame, neohesperidin dihydrochalcone, neotame, and steviol glycoside	Alcoholic and non-alcoholic beverages, and three instant drink powders	HILIC–MS/MS	AcclaimTM TrinityTM P2 (100 mm × 2.1 mm, 3 µm); mobile phase A: acetonitrile with 0.01% acetic acid; mobile phase B: 10 mM of ammonium acetate; gradient elution; flow rate: 0.6 mL/min; injection volume: 2 µL	Buffer 40 mM (pH 6.8) can speed up the analysis time and sharpens peaks; high trueness and repeatability; simple sample preparation	A higher buffer concentration (more than 40 mM) could causes a decrease in detection sensitivity; analysis time with HILIC was longer than RPLC	Recovery: 98.6–106.2% LOD: 0.00018–0.033 mg/L LOQ: 0.0023–0.01 mg/L	[31]
Acesulfame, aspartame, neo-hesperidin dihydrochalcone, neotame, and saccharin	Soft and powdered drinks, juices, teas, soy drinks, dairy-based drinks, beers, and spirit	UHPLC–PDA	Kinetex C18 column (50 mm × 2.1 mm, 1.7 mm); flow rate of 0.3 mL/min; mobile phase A: acetonitrile; mobile phase B: phosphate buffer pH 6 (1 mmol/L); gradient elution; column temperature: 30 °C	High recovery; rapid analysis time (3 min); reduced solvent consumption; better sensitivity and resolution than HPLC	Cyclamate cannot be detected by PDA because it does not have UV absorption	Recovery: 90–114.6% LOD: N/A LOQ: 0.01–0.1 mg/L	[74]
		HPTLC (for sucralose)	Pre-coated silica gel ^60^F_254_ (20 cm × 10 cm); mobile phase: acetonitrile: water (16:4, *v*/*v*); scanned at: 366 nm.				
Acesulfame, saccharin, aspartame, stevioside, and neotame	Wine	HPLC–UV	C18 column (250 mm × 4.6 mm, 5 μm); mobile phase A: 2.5 mmol/L AmAc and 0.01% TFA in water; mobile phase B: acetonitrile; gradient elution; column temperature: 30 °C; flow rate: 1 mL/min; detection wavelength: 210 nm; injection volume: 10 μL	A clean-up step prior to RP-HPLC–UV provides excellent results as reducing the interferences from the complex matrix	The chromatographic analysis time was 20 min	Recovery: 80.1–97% LOD: 0.12–0.31 mg/L LOQ: 0.35–0.92 mg/L	[75]
Acesulfame, cyclamate, saccharin, aspartame, alitame, neotame, sucralose, and stevioside	Wine, beers, orange juices, apple juices, herbal tea, candied fruits, canned peaches, canned mangos, and cakes	HPLC/ESI–MS	C18 silica (250 mm × 4.5 mm i.d., 5 μm); buffer solution: formic acid:triethylamine (0.8:1.5, *v*/*v*) in 1 L of water; mobile phase A: methanol:buffer solution: acetone (69:24:7, *v*/*v*/*v*); mobile phase B: methanol:buffer solution: acetone (11:82:7, *v*/*v*/*v*); gradient elution; flow rate: 1 mL/min; injection volume: 10 μL	High recovery and sensitivity; the addition of acetone to the mobile phase can increase the ionization efficiency; with the composition of the mobile phase used can reduce ion suppression by the sample matrix	Sample preparation without the purification step can increase ion suppression in the ESI. One of the causes is the presence of endogenous substances in the extract sample so that a proper sample preparation protocol is needed	Recovery: 95.4–104.3% LOD: 0.01–0.10 mg/L LOQ: 0.03–0.30 mg/L	[76]
Acesulfame, aspartame, neohesperidin dihydrochalcone, and saccharin	Candies, jellies, and beverages	Normal-phase HPTLC	Aluminum-backed HPTLC plates (10 × 10 cm) pre-coated with silica gel F_254_; mobile phase: acetonitrile: water: ethyl acetate: 10% aqueous ammonia (9:1:1:1, *v*/*v*/*v*/*v*); HPTLC scanned on: deuterium lamp; scan rate: 20 mm/s; and λ: 210, 295, 450, 550 nm	Low-cost; high recovery; selectivity is acceptable, marked by no interference from organic acids and sugars	The working conditions is more complicated than the HPLC method in general	Recovery: 96.6–106.7% LOD: N/A LOQ: N/A	[77]
Acesulfame, alitame, aspartame, cyclamate, neotame, neohesperidin, dihydrochalcone, saccharin, and sucralose	Beverages	RP-HTLC–MS/MS	Shodex ET-RP1 column (150 mm × 3.0 mm, 4 µm); gradient elution: mobile phase A: water with 5 mM ammonium acetate; mobile phase B: ethanol; flow rate: 0.4 mL/min; temperature gradient: 0–9 min: 110–150 °C at a rate of 8 °C/min, hold 6 min, and lowered back to 110 °C for column re-equilibration; injection volume: 10 μL	Considered as green chromatography analysis because of the use of non-toxic solvents such as water and a small amount of ethanol (no more than 1 mL per sample)	The chromatographic analysis time was 20 min, including the column equilibrium process; due to the water-based mobile phase, very high temperature can cause hydrolysis between the silica and analyte bonds.	Recovery: 86–110% LOD: 0.05–10 mg/L LOQ: 0.17–33 mg/L	[78]
Aspartame and its thermal hydrolysis and racemization products, and amino acid enantiomers	Cola and sugar free cola	Two-dimensional HPLC Fluorescent-LEC	Column temperature: 50 °C; mobile phase: 2 mM CuSO_4_/methanol (80:20, *v*/*v*); flow rate: 1 mL/min; First dimension: RP Zorbax Eclipse XDB-C8 (150 mm × 4.6 mm, 5 µm) and Zorbax Eclipse XDB-C8 guard column (12.5 mm × 4.6 mm, 5 µm); Ultraviolet detector at λ = 254 nm. Second dimension: ligand-exchange column (LEC), Chirex 3126d-penicillamine column (250 mm × 4.6 mm, 5 µm); fluorescence detection at λ: 340 nm and 450 nm.	This technique is designed for the simultaneous analysis of aspartame and its hydrolyzed products (amino acids such as aspartic acid and phenylalanine); fluorescence detection provides better sensitivity than UV	Time-consuming separation (almost 1 h)	Recovery: 90.2–99.2% LOD: 1.3 mg/L LOQ: 4.3 mg/L	[22]
Acesulfame and saccharin	Cola, grape soda, sprite, orange soda, green tea, black tea, orange juice, apple juice, milk drink, grape wine	ATLD-assisted HPLC–DAD	WondaSil C18 reversed-phase column (200 mm × 4.6 mm, 5 μm); mobile phase A: water mixed with 20 mmol/l ammonium acetate; mobile phase B: acetonitrile; gradient elution; flow rate: 1 mL/min; DAD scan at λ: 190–800 nm with a step of 1.2 nm; acquisition rate: 0.64 s/cycle; injection volume: 20 µL	Overlapping peak and baseline drift can be overcome with a second-order calibration method based on an alternating trilinear decomposition (ATLD) algorithm.	The lack of pretreatment can lead to baseline drift, interference from unknown analytes, and overlapped (especially for complex matrices)	Recovery: 87.3–103 LOD: 0.0014–0.165 mg/L LOQ: 0.0042–0.5 mg/L	[79]
Acesulfame, cyclamate, saccharin, aspartame, sucralose, neohesperidin dihydrochalcone, and neotame	Wastewater, tap water, surface water (including river water and seawater), and groundwater	Ion-pair LC–MS/MS	Athena C18-WP column (4.6 mm × 150 mm, 3 µm); column temperature: 30 °C; mobile phase A: water; mobile phase B: acetonitrile, both containing 5 mM ammonium acetate and 1 mM TRIS; flow rate: 0.4 mL/min; gradient elution; injection volume: 20 µL; separation time: less than 13 min	High reproducibility and sensitivity	Extraction recovery for aspartame is less than 80%	Recovery: 79–116% LOD: 0.1–2.3 ng/L LOQ: 0.4–7.5 ng/L	[37]
Acesulfame, alitame, aspartame, cyclamate, neotame, neohesperidin dihydrochalcone, saccharin, stevioside, and sucralose	Fish	LC–MS	Ascentis Express RP amide (100 mm × 2.1 mm, 2.7 µm) and Zorbax Eclipse XDB-C8 (150 mm × 4.6 mm, 5 µm); mobile phase A: ultrapure water at pH 2.5 with formic acid; mobile phase B: acetonitrile; gradient elution; column temperature: 25 °C; injection volume: 25 µL; total separation time: 15 min	The extraction method permits a small amount of sample with a rapid extraction time (5 min)	Cyclamate and saccharin showed poor fragmentation compared to other analytes; low recovery for neohesperidin dihydrochalcone	Recovery: 46–94% LOD: 0.0025–0.125 mg/L LOQ: 0.0125–0.25 mg/L	[12]
Advantame and neotame	Ham, snack confections, jelly	LC–MS/MS	Acquity UPLC CSH C18 column (100 mm × 2.1 mm, 1.7 µm); mobile phase A: 10 mmol/L ammonium formate; mobile phase B: methanol; gradient elution; flow rate: 0.2 mL/min up to 8 min and 0.5 mL/min from 8.1 to 10 min; injection volume: 3 µL; ion-source temperature: 300 °C	High sensitivity and accuracy; without clean-up step (SPE); fast separation (total run time: 10 min)	Requires a complicated and time-consuming extraction (1 h)	Recovery: 76.1–102.7% LOD: <0.01 mg/L LOQ: 0.01 mg/L	[80]
Aspartame, cyclamate, acesulfame, and saccharin	Carbonated cola drinks and fruit juice drink	IC	Ionpac AG11 guard column (50 mm × 2 mm) and a Dionex Ionpac AS11 Separation column (250 mm × 2 mm); temperature: 35 °C; flow rate: 0.25 mL/min; injection volume: 25 μL	High sensitivity and reproducibility; no interference from organic or inorganic ions	There is no reference method to validate the proposed method	Recovery: 98–105% LOD: 0.019–0.87 mg/L LOQ: N/A	[81]
Sucralose, cyclamate, acesulfame, and saccharin	Drinking water, groundwater, surface water, and domestic wastewater	IC–MS/MS	IONPAC AS19 column (150 mm × 2 mm); flow rate of 0.3 mL/min; mobile phase: 60 mM sodium hydroxide; isocratic elution; ion source temperature: 600 °C and ion spray voltage: −3500 V	High sensitivity; enhancement of analyte separation without the addition of ion-pair reagents; fast analysis (total run time: 9 min)	A high ion temperature source is required to improve the ionization efficiency	Recovery: 65–120% LOD: 1.7–12.5 mg/L LOQ: N/A	[82]
Aspartame, saccharine, and sucralose	Water, soft drinks, liquid syrups	LC–TOF/MS	RP C8 analytical column (150 mm × 4.6 mm, 5 µm); flow rate: 0.6 mL/min; mobile phases A: acetonitrile with 0.1% formic acid; mobile phases B: water with 0.1% formic acid; gradient elution; injection volume 50 µL	The derivatization process is not required	Sucralose fragmentation resulting in broad peaks; saccharin has very low recovery	Recovery: N/A LOD: 0.005–0.1 mg/L LOQ: 0.05–1 mg/L	[83]
Cyclamate	Soft drinks and sweetener tablets	GC–FID	CPBS fused-silica capillary column (25 m × 0.22 mm, 0.25 µm); flow rate: 1 mL/min of nitrogen; oven temperature: 55–60 °C at a rate of 30 °C/min for 1 min and increased to 230 °C at a rate of 40 °C/min for 0.5 min; detector temperature: 200 °C	High recovery; HS-SDME was used as an alternative of simpler derivatization technique compared to conventional derivatization [29]	Requires derivatization	Recovery: 96.6–97.6% LOD: 0.5757 mg/L LOQ: N/A	[34]
Saccharin and cyclamate	Tabletop sweeteners	FT-Raman spectroscopy	Spectra were recorded between 3500 and 75 cm^−1^, with a resolution at 4 cm^−1^ accumulating 64 scans per spectrum; laser power at 250 mW; a scan velocity of 2.2 kHz, a zero filling factor of 2, and an aperture of 10 mm	Non-destructive method; eliminates the use of reagent and solvent	Low sensitivity than HPLC for sodium saccharin and sodium cyclamate in tabletop sweeteners	N/A LOD: 2000–8000 mg/L LOQ: N/A	[67]
Cyclamate, sucralose, saccharin, acesulfame, and aspartame	Diet tea drinks	FTIR	Frontier Optica FTIR; wavelength: 4000–400 cm^−1^ (infrared spectra for analytes from 1500 to 1000 cm^−1^); resolution: 0.4 cm^−1^; and 20 scans per sample	Non-destructive technique; sample pretreatment is not required; fast detection and eliminating the use of solvent	Before analysis, correction step was needed to reduce interference and noise	Recovery: 94% LOD: N/A LOQ: N/A	[66]
Aspartame, saccharin, and acesulfame	Liquid diet-drink and commercial sweetener pills	Double-beam UV–Vis Spectrophotometer and LC	Double-beam UV–Vis Spectrophotometer equipped with a 1 cm quartz cell; spectra recording at 200–300 nm	Multivariate standard addition method based on net analyte signal concept (SANAS) can be used to overcome the interference, either directly or indirectly	The selection of pH using Clark–Lubs buffer is very influential in the analysis. The use of high pH (>10) gives good sensitivity but not selectivity	Recovery: 97.4–108.4% LOD: 0.05–0.21 mg/L LOQ: 0.15–0.68 mg/L	[84]
Aspartame, potassium acesulfame, and saccharine	Dehydrated soups and soft drink	FIA	Monolithic column C18 (5 mm × 4.6 mm); sample volume: 125 µL; carrier A: 4% acetonitrile 10 mM phosphate buffer pH 6.0; flow rate of 3.5 mL/min; carrier B: 30% methanol in water; separation time: 400 s	Rapid; simple; low-cost; high repeatability and reproducibility; and good resolution	Compare with HPLC (as a reference method), HPLC has a better resolution than FIA	Recovery: 96.8–101.5% LOD: 0.01–0.73 mg/L LOQ: 0.94–2.43 mg/L	[85]
Aspartame, cyclamate, acesulfame, and saccharin	Soft drinks, liquid and solid sweeteners, peach tea, lemon tea, syrup	CZE with indirect UV detection	An uncoated fused-silica capillary (400 mm × 50 µm); UV detection at 220 nm; electroosmotic flow (EOF) maker: acetone; pressure injection for a mixture of sample and EOF: 50 mbar/5 s	Rapid separation (less than 1 min); high selectivity and robust	Co-ions such as benzoic acid is required to form chromophores because there is no UV absorption for cyclamate	Recovery: 91–117% LOD: 3.3–6.4 mg/L LOQ: 9.4–21.4 mg/L	[42]
Acesulfame, aspartame, and saccharin	Soft drinks	CZE and MEKC–DAD	Uncoated fused-silica capillary (48.5 cm × 50 µm); absorbance was measured at 200 nm; hydrodynamic injection: 250 Mbars; capillary temperature at 25 °C; separation voltage at 20 kV; micellar agent: sodium dodecyl sulfate (SDS)	The use of micelle agents provides acceptable separation	Low resolution due to the presence of interference.	Recovery: N/A LOD: 0.35–2.12 mg/L LOQ: 10 mg/L	[86]
Sucrose, saccharin, and cyclamate	Cola and free sugar cola	Epithelium biosensor	Isolated epithelium (about 5 mm × 5 mm) from rats; rinse solution: oxygenated Ringer’s solution; flow rate: 1 mL/min; signal recorded by microelectrode array (MEA) (MEA1060-Inv system from Multichannel Systems) with 60 electrodes (30 μm in diameter with 200 μm center to center spacing); temperature: 25 °C.	Rapid detection; good reproducibility; the ability to distinguish between sweeteners and sugars, also analytes that have the same functional group	Less effective for analysis of more than two types of sweeteners	Recovery, LOD, and LOQ: N/A	[87]

FIA, flow-injection analysis; LC/TOF-MS, Liquid Chromatography/Time-of-Flight Mass Spectrometry; HILIC, Hydrophilic-Interaction Liquid Chromatography; HTLC–MS/MS, High-Temperature Liquid Chromatography–Tandem Mass Spectrometry; HPTLC, High-Performance Thin-Layer Chromatography; CE, capillary electrophoresis; CZE, capillary zone electrophoresis; HS-SDME, headspace single-drop microextraction; TEPA-MP, tetraethylenepentamine-functionalized Fe_3_O_4_ magnetic polymer; dSPE, dispersive solid-phase extraction.
